# Investigation into Safety Profiles of Antiepileptic Drugs and Identification of Predictors for Serious Adverse Events: Insights from National Pharmacovigilance Data

**DOI:** 10.3390/ph18071013

**Published:** 2025-07-07

**Authors:** Soo Hyeon Lee, Dae Hyeon Sung, Euna Cho, Jeongah Min, Sooyoung Shin, Yeo Jin Choi

**Affiliations:** 1 Department of Regulatory Science, Graduate School, Kyung Hee University, Seoul 02447, Republic of Korea; 2 Institute of Regulatory Innovation through Science (IRIS), Kyung Hee University, Seoul 02447, Republic of Korea; 3 Department of Pharmacy, School of Pharmacy, Kyung Hee University, Seoul 02447, Republic of Korea; 4 Research Institute of Pharmaceutical Science and Technology (RIPST), Ajou University, Suwon 16499, Republic of Korea; 5 Department of Pharmacy, College of Pharmacy, Ajou University, Suwon 16499, Republic of Korea

**Keywords:** anti-epileptic drugs, geriatrics, pharmacovigilance, psychotropic drugs, opioids, acid suppressive therapy, drug–drug interactions

## Abstract

**Backgrounds/Objectives:** This study aims to comprehensively characterize the prevalence and severity of antiepileptic drug (AED)-induced adverse drug events (ADEs) and to identify predictors strongly associated with serious adverse events (SAEs) in both general and geriatric populations. **Methods:** This cross-sectional study investigated AED-related ADEs reported to the KIDS KAERS DB from January 2014 to December 2023. Disproportionality analysis was performed to detect the association between reported SAEs, and multiple logistic regression was conducted to identify predictors associated with SAEs. Cox’s proportional hazard model was utilized to assess ADE duration in elderly patients aged 60 years and older. **Results:** More than 50% of 36,809 AED-related ADEs were reported in elderly patients aged 60 years and older, and the prevalence of SAEs was 3.78%. ADEs associated with endocrine disorders had the highest likelihood of SAEs being reported (ROR 15.30), followed by hematological disorders. The predictors associated with elevated SAE risks in the elderly were male sex (OR 1.91; 95% CI 1.62–2.27), aging (OR 1.17; 95% CI 1.04–1.31), and certain AEDs. However, the concomitant administration of acid-suppressive therapy (AST) and opioids was associated with a lower risk of SAEs in the elderly population. Elderly patients not receiving concomitant AST were less likely to experience prolonged ADE duration (HR 0.28, 95% CI 0.07–1.15); however, no substantial differences in ADE duration were observed with the concomitant use of opioids. **Conclusions:** This study implies significant variability in the frequency, severity, and duration of ADEs depending on the type of AEDs, patient demographics, and concomitant medication use.

## 1. Introduction

Epilepsy is a brain disorder characterized by an increased predisposition to epileptic seizures, leading to transient occurrences of signs and/or symptoms associated with abnormal or excessive neuronal activity in the brain [1]. Epilepsy is associated with numerous neurological, cognitive, psychological, and social consequences. The global prevalence of epilepsy is increasing, affecting 51.7 million patients, with an age-standardized prevalence rate of 657 per 100,000 patients in 2021 [1,2]. The management of epilepsy remains a significant clinical challenge despite the numerous antiepileptic drugs (AEDs) available on the market, and a recent study indeed reported a prevalence of drug-refractory epilepsy of 21.3% [3,4]. Moreover, the frequent occurrence of numerous adverse drug events (ADEs) associated with AEDs complicates epilepsy management as well [3]. These ADEs may range in severity from mild to severe, or even life-threatening, which substantially affects patient adherence to the treatment and overall quality of life [5].

One of the most significant challenges in epilepsy management is the risk of drug–drug interactions (DDIs) associated with AEDs, particularly the first generation of AEDs, such as carbamazepine and phenytoin [5]. Many AEDs either induce or inhibit the cytochrome P450 (CYP450) enzyme, subsequently increasing the risk of potential drug–drug interactions and ADEs [5]. Furthermore, AEDs themselves may interact with one another, and this is especially concerning for patients with refractory epilepsy, who often require multiple AEDs concurrently to manage epilepsy. Moreover, these patients are predisposed to an elevated ADE risk due to the altered pharmacokinetic properties and reduced therapeutic efficacy associated with potential drug–drug interactions.

Geriatric populations, defined as the population aged 65 years and older, are at an elevated risk of severe adverse events (SAEs) due to altered pharmacokinetic/pharmacodynamic characteristics, polypharmacy, and multimorbidity [6]. They also have an elevated risk of cognitive impairment, falls, frailty, and comorbidities such as cardiovascular and endocrinology disorders, all of which may be exacerbated by AED-induced ADEs, further complicating epilepsy management [7]. Furthermore, age-related physiological changes may further exacerbate the pharmacokinetic/pharmacodynamic impact of AEDs, increasing the vulnerability of geriatric patients to ADEs [8]. Nonetheless, the impact of AED-induced ADEs in geriatric patients is limited despite their heightened risk. Moreover, considering the substantial increase in the aging population worldwide, a comprehensive investigation into the safety of AEDs as well as the identification of predictors associated with SAEs in the geriatric population is essential. Thus, the aim of this study was to comprehensively characterize the prevalence and severity of AED-induced ADEs and to identify predictors strongly associated with SAEs in both general and geriatric populations by utilizing a spontaneous adverse event system to promote drug safety in patients with epilepsy.

## 2. Results

### 2.1. Patient Demographics

**Figure 1:**
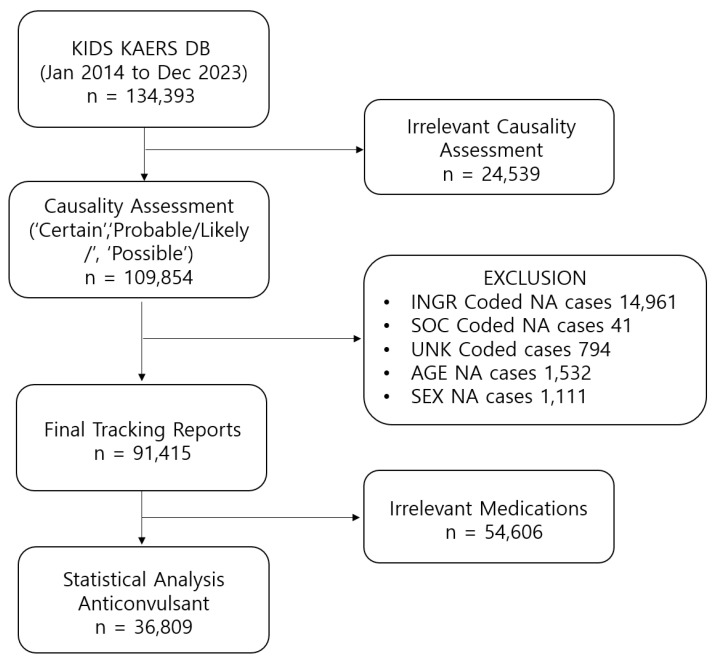
Data acquisition process.

Among 134,393 ADE cases extracted from the Korean Adverse Event Reporting System database (KIDS KAERS DB), a total of 36,809 AED-related ADEs reported from 1 January 2014, to 31 December 2023, were included in the analysis (Figure 1). The highest number of ADEs was reported in elderly patients aged 60 years and older (N = 19,155; 52.04%) (Table 1). Approximately 65.62% of cases were reported in women (N = 24,153). The prevalence of SAEs was 3.78% (N = 1392). The most etiologic AEDs were pregabalin (N = 12,318; 33.46%) and gabapentin (N = 11,062; 30.05%). The highest likelihood of SAEs being reported was observed for valproic acid (reporting odds ratio (ROR) 6.18, 95% confidence interval (CI) 4.79–7.98), followed by phenytoin (ROR 4.03, 95% CI 3.09–5.25) and phenobarbital (ROR 3.86, 95% CI 2.43–6.13) (Table 2). The likelihood of SAEs being reported was substantially lower for gabapentin (ROR 0.20, 95% CI 0.16–0.24) and pregabalin (ROR 0.36, 95% CI 0.31–0.41).

### 2.2. ADE Types and Risk of Reporting SAEs

The most frequent types of AED-related ADEs were associated with central and peripheral nervous system disorders (N = 8270; 22.47%) and respiratory system disorders (N = 7976; 21.67%) (Table 3). However, reported ADEs associated with endocrine disorders had the highest likelihood of being SAEs (ROR 15.30; 95% CI 3.65–64.07), followed by hematological disorders, including red blood cell disorders (ROR 10.01; 95% CI 4.63–21.68) and white cell and reticuloendothelial system (RES) disorders (ROR 9.00; 95% CI 6.90–11.72). The odds of reporting SAEs were substantially lower among ADEs associated with central and peripheral nervous system disorders (ROR 0.41; 95% CI 0.35–0.49), psychiatric disorders (ROR 0.20; 95% CI 0.15–0.28), and gastrointestinal system disorders (ROR 0.06; 95% CI 0.03–0.10). The results of the disproportionality analysis of SOC-based ADEs for individual AEDs are summarized in Table 4. The highest likelihood of reporting both serious and nonserious skin and appendages disorder was observed for lamotrigine (ROR 12.46, 95% CI 11.15–13.92 for non-SAEs, and ROR 3.30 95% CI 2.40–4.55 for SAEs), followed by phenytoin (ROR 5.18, 95% CI 4.26–6.29 for non-SAEs and ROR 2.73, 95% CI 1.66–4.50 for SAEs) and carbamazepine (ROR 5.10, 95% CI 4.61–5.64 for non-SAEs and ROR 2.17, 95% CI 1.64–2.87 for SAEs). Interestingly, levetiracetam was more likely to lead to reports of nonserious skin and appendages disorder (ROR 2.42, 95% CI 2.19–2.68) than serious cases, where the signal was inversely associated with this risk (ROR 0.40, 95% CI 0.27–0.61). Valproic acids and clonazepam are more likely to be associated with serious central and peripheral nervous system disorders than nonserious cases. Topiramate was the only agent with a substantially high likelihood of serious vision-related ADEs being reported (ROR 54.45 (95% CI 13.23–224.12). Clonazepam and topiramate had a markedly high likelihood of both serious and nonserious ADEs related to psychiatric disorders being reported, whereas levetiracetam and phenytoin were more likely to lead to reports of both nonserious and serious liver and biliary disorders. Oxcarbamazepine had a significantly high likelihood of both serious and nonserious ADEs related to metabolic disorders and hematologic disorders being reported, including red blood cell disorders and white cell and reticuloendothelial system (RES) disorders. The odds of reporting SAEs were substantially higher with concomitant chemotherapy administration (ROR 1.93, 95% CI 1.02–3.69) (Table 5). However, the concomitant administration of acid-suppression therapy (AST), acetaminophen, non-steroidal anti-inflammatory drugs (NSAIDs), opioids, antidepressants, and antihyperglycemic agents was associated with a lower likelihood of SAEs being reported.

### 2.3. ADE Types and Risk of SAE Reporting in Elderly Patients

The highest likelihood of AED-related SAEs reported in elderly patients was observed for white cell and RES disorders (ROR 10. 78; 95% CI 7.08–16.41), followed by liver and biliary system disorders (ROR 6.25; 95% CI 4.25–9.21) and cardiovascular disorders (ROR 3.07; 95% CI 1.32–7.13) (Table 6). In contrast, the likelihood of reporting SAEs was lower with central and peripheral nervous system disorders (ROR 0.45; 95% CI 0.35–0.57), psychiatric disorders (ROR 0.23; 95% CI 0.15–0.37), and gastrointestinal disorders (ROR 0.03; 95% CI 0.00–0.09). The results of the disproportionality analysis of SOC-based ADEs for individual AEDs in elderly patients are summarized in Table S1. The concomitant use of AST, acetaminophen, NSAIDs, opioids, and antidepressants was associated with a lower likelihood of SAEs being reported (Table 7).

### 2.4. Identification of Predictors Associated with SAE Risks

Univariate analysis identified patient sex, causality, the number and types of concomitant medications, and AED types as predictors associated with SAEs (Table 8). Multivariate logistic regression revealed a significantly increased risk of SAEs with male sex (OR 1.36; 95% CI 1.21–1.52), concomitant immunosuppressant (OR 3.68; 95% CI 1.39–9.74) and chemotherapy (OR 2.22; 95% CI 1.11–4.45) use, and certain AEDs, including divalproex, lamotrigine, levetiracetam, valproic acid, oxcarbazepine, zonisamide, carbamazepine, phenobarbital, and phenytoin. The predictors associated with elevated SAE risks in the elderly were male sex (OR 1.91; 95% CI 1.62–2.27), aging (OR 1.17; 95% CI 1.04–1.31), and certain AEDs, including lamotrigine (OR 2.26; 95% CI 1.43–3.56), levetiracetam (OR 1.83, 1.25–2.69), valproic acid (OR 3.70; 95% CI 1.96–7.00), carbamazepine (OR 2.78, 95% CI 1.94–3.98), and phenytoin (OR 3.33; 95% CI 1.98–5.58) (Table 9). However, the concomitant administration of AST, opioids, and antidepressants was associated with a lower risk of SAEs in elderly patients.

### 2.5. Impact of SOC on ADE Duration in the Elderly

**Figure 2:**
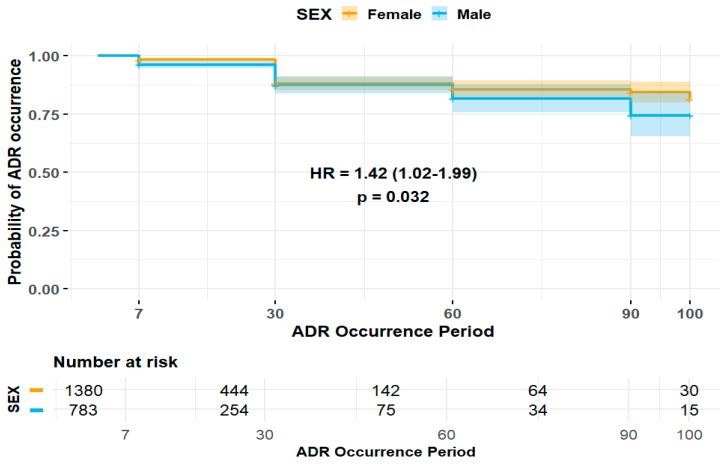
Impact of sex on duration of ADEs in elderly patients.

**Figure 3:**
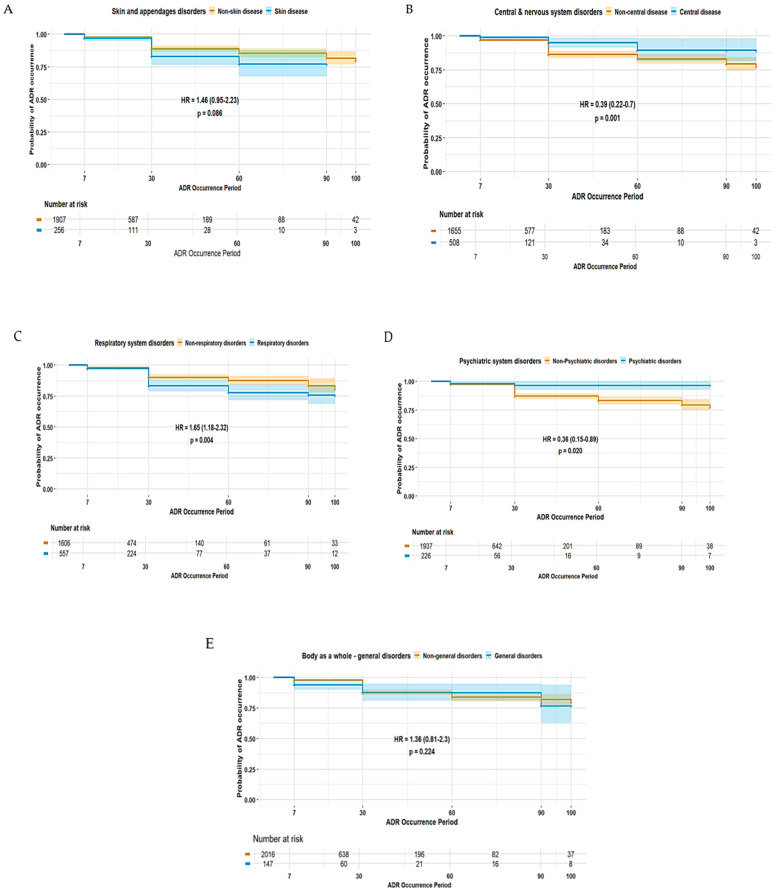
Duration of ADEs associated with SOC-based classifications in elderly patients: (**A**) skin and appendages disorders; (**B**) central and peripheral nervous system disorders; (**C**) respiratory system disorders; (**D**) psychiatric disorders; and (**E**) disorders of the body as a whole.

**Figure 4:**
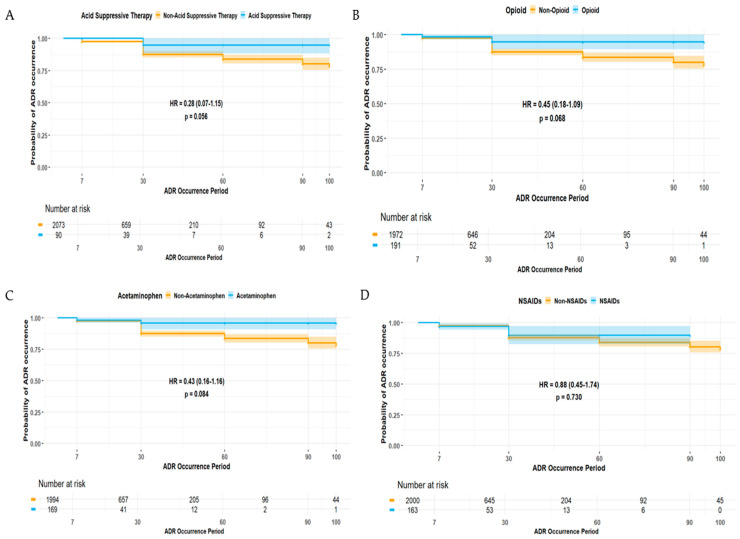
Duration of ADEs associated with types of concomitant medications: (**A**) acid-suppressive therapy (AST), (**B**) opioids, (**C**) acetaminophen, and (**D**) non-steroidal anti-inflammatory drugs (NSAIDs).

The duration of ADE was significantly longer in female patients (HR 1.42, 95% CI 1.02–1.99) (Figure 2). Patients experiencing ADEs unrelated to the central and peripheral nervous system (HR 0.39, 95% CI 0.22–0.70) and non-psychiatric disorders (HR 0.36, 95% CI 0.15–0.89) were less likely to have prolonged ADE duration, whereas those patients experiencing ADEs related to non-respiratory disorders were more likely to experience a longer duration of ADEs (HR 1.65, 95% CI 1.18–2.32) (Figure 3). Among elderly patients, those not receiving concomitant AST were less likely to experience prolonged ADE duration (HR 0.28, 95% CI 0.07–1.15) (Figure 4). No substantial differences in ADE duration were observed with the concomitant use of opioids, acetaminophen, and NSASIDs.

## 3. Discussion

This study comprehensively evaluated AED-induced ADE records that were spontaneously reported to a nationwide pharmacovigilance reporting system, KIDS KAERS DB, from 1 January 2014, to 31 December 2023. This study demonstrated that the majority of the ADEs were reported in the geriatric population aged 60 years and older, implying the increased vulnerability of older adults to drug-related ADEs and complications (Table 1). Among numerous AEDs, gabapentin and pregabalin were the most frequently reported agents; however, both agents were associated with a substantially lower risk of SAEs being reported, suggesting a favorable safety profile compared to older-generation AEDs, such as phenytoin and phenobarbital (Table 2). The most frequently reported types of ADEs were those associated with central and peripheral nervous system disorders and respiratory system disorders, whereas the highest likelihood of SAEs being reported was observed for ADEs related to endocrine disorders, hematologic disorders, and liver and biliary disorders (Table 3). The predictors associated with elevated AED-induced SAEs were male sex, the type of AEDs, and the concomitant use of immunosuppressants and chemotherapy (Table 8). However, increasing age was an essential predictor for elevated SAE risk in the elderly population (Table 9).

The risk of SAEs is substantially higher for skin and appendages disorder, liver and biliary disorders, respiratory system disorders, and white cell RES disorders in patients from both the general and geriatric population (Table 3 and Table 6). Severe skin and appendages ADEs, such as Stevens–Johnson syndrome (SJS), drug reactions with eosinophilia and systemic symptoms (DRESS), and toxic epidermal necrolysis (TEN), are well-recognized as being rare, but there are serious complications for certain AEDs, particularly carbamazepine, lamotrigine, and valproic acids [9,10]. Consistent with previous findings, this study demonstrated the substantially elevated likelihood of both nonserious and serious skin and appendages disorder being reported in relation to the ADEs of lamotrigine and carbamazepine (Table 4). These reactions often require intensive medical interventions and substantially increase morbidity, especially in older adults [11]. Moreover, evidence suggests a higher risk of drug-induced skin-related ADEs in Asian populations, which may be partially explained by genetic susceptibility, such as the HLA–B*15:02 alleles [12]. Furthermore, the rapid titration of an AED dose also contributes to an elevated risk of skin-related reactions in epilepsy patients, implying the importance of pharmacogenetic screening and optimal dose titration in Asian elderly populations [13].

Drug-induced liver and biliary injury is also a well-recognized complication of AEDs, primarily due to their hepatic metabolism and hepatotoxic potentials. Several AEDs, including valproic acids, phenytoin, and carbamazepine, are extensively metabolized by the liver and have been associated with both idiosyncratic and dose-dependent liver injuries, ranging from an asymptomatic elevation in liver enzymes, such as aspartate transaminase (AST) and alanine transaminase (ALT), to fulminant hepatic failure [14]. Furthermore, these agents exhibit potent modulatory activity, functioning either as an inducer or inhibitor, on the CYP450 enzyme system, subsequently contributing to clinically significant DDIs [15]. These pharmacokinetic interactions are particularly critical in geriatric patients, who often experience an age-related reduction in hepatic clearance and polypharmacy, increasing their susceptibility to liver injury and ADEs resulting from significant DDIs. Hence, a comprehensive medication review and liver function monitoring are critical to mitigate the risk of severe liver-related ADEs.

ADEs involving the central and peripheral nervous system were among the most frequently reported ADEs in this study (Table 3). These ADEs typically include symptoms such as somnolence, tremor, peripheral neuropathy, and cognitive impairments, many of which are dose-dependent and may significantly affect daily functioning [16]. Despite their lower likelihood of being reported as SAEs, elderly patients experiencing central and peripheral nervous systems-related ADEs are more likely to have prolonged event duration (Figure 3), implying that the burden of central and peripheral nervous system-related ADEs is not necessarily reflected by severity alone but also by their persistence and functional impact over time. The chronic or slowly resolving nature of many neurologic side effects, in addition to delayed recognition or insufficient dose adjustment, may contribute to extended symptom duration. Moreover, elderly patients are particularly vulnerable to AED-induced cognitive impairment due to age-related declines in neuroplasticity, and these patients often experience slower recovery from neurologic toxicity [17,18]. Moreover, in this study, ADEs related to white cell and RES system disorders were associated with a significantly increased likelihood of SAEs being reported (Table 3). Several AEDs, including carbamazepine, phenytoin, valproic acid, and phenobarbital, have been implicated in a range of hematologic toxicities, such as leukopenia, aplastic anemia, and thrombocytopenia [19], and this study provided consistent findings (Table 4). Again, these ADEs can either be idiosyncratic or dose-related, especially in the case of prolonged exposure or high plasma concentrations [20]. This risk is more pronounced in elderly patients, who may have reduced bone marrow reserves or altered drug metabolism [21,22]. Hence, this study emphasizes the need for cautious neurologic and hematologic monitoring, individualized dosing strategies, and early intervention to minimize neurologic ADEs in aging populations.

In this study, gabapentin and pregabalin emerged as the most frequently reported etiologic AEDs (Table 2), and this may reflect their widespread use as adjunctive treatments, not only in refractory epilepsy but also in neuropathic pain, fibromyalgia, and anxiety disorders, especially in older adults [23,24]. Although their frequent use contributed to the high number of reported ADEs, both gabapentin and pregabalin were associated with a substantially lower likelihood of SAEs being reported, implying a generally favorable safety profile compared to older-generation AEDs. However, these agents may induce central nervous system (CNS)-related ADEs, including somnolence, dizziness, ataxia, and cognitive impairment, particularly among elderly patients or those receiving polypharmacy. Elderly patients are at a higher risk of pain from multiple comorbidities, including osteoarthritis and diabetic neuropathy, which may lead to the concomitant use of other analgesics, such as acetaminophen, NSAIDs, and opioids [25]. Interestingly, this study found that the concomitant use of opioids, NSAIDs, and acetaminophen was associated with a lower likelihood of SAEs being reported (Table 5 and Table 7) and an insignificantly prolonged duration of ADEs (Figure 4). This may result from cautions for prescription practices: short-term or low-dose use in high-risk patients. Nonetheless, caution is warranted, particularly with gabapentinoid–opioid combinations, as previous research has revealed a markedly increased risk of respiratory depression and overdose [26]. These findings accentuate the importance of individualized treatment plans and close monitoring for ADEs related to disorders of the central nervous system and respiratory system in elderly patients.

Another interesting finding was prolonged ADE recovery among patients receiving concomitant AST despite its association with a lower risk of SAEs (Figure 4). This may be associated with altered pharmacokinetic characteristics by histamine-2 receptor antagonists (H2RAs) and proton pump inhibitors (PPIs). These agents alter gastric pH, which can contribute to altered absorption and bioavailability of certain AEDs, especially those with pH-dependent solubility [27]. Interestingly, a study revealed that the long-term use of PPIs has been linked to a substantially increased risk of developing epilepsy [28]. While the mechanism behind this remains unclear, disruption of the gut–brain axis, micronutrient deficiencies, and chronic system inflammation induced by altered gastrointestinal microbiota may have a role in inducing epilepsy [28]. These findings suggest that prolonged AST use may have neurological implications other than pharmacokinetic interactions, implying the importance of judicious AST prescription, especially in patients with epilepsy or those receiving long-term AED therapy. Moreover, further controlled studies are warranted to elucidate the clinical significance of AST-related neurologic risks and their impact on central nervous system outcomes in epilepsy management.

This study provided a comprehensive pharmacovigilance investigation of AEDs, including detailed insights into the types and duration of AED-induced ADEs as well as their prevalence and severity over a 10-year period. This study also provides differentiated analyses by age group, particularly focusing on elderly patients, who are often underrepresented in clinical trials but are predisposed to an elevated risk of drug-related problems and complications. Furthermore, this study investigated the impact of concurrent medication therapy with AEDs on ADE severity and types. Nonetheless, this study possesses several limitations. First, KIDS KAERS DB is a spontaneous voluntary ADE reporting system, which provides nationwide and longitudinal insights into drug-induced ADEs, but it is subject to potential issues with reporting bias, underreporting, and variability in data completeness and quality. The absence of comprehensive patient histories, including comorbidities, concurrent medications, treatment duration, and exact medication dosages, may hinder the accurate determination of the causality between AED exposure and reported ADEs, thereby leading to an underestimation or misinterpretation of the risk factors contributing to AED-induced ADEs. Moreover, potentially important clinical and demographic factors—including medication adherence, body mass index (BMI), education level, and socioeconomic status—were not available in the KIDS KAERS DB, limiting the analysis to evaluating potential correlations between the variables and the occurrence of ADEs with different severities. Additionally, certain drugs, such as oxycodone and other opioids, were excluded from the subset used for the disproportionality analysis due to a low number of SAE reports, not meeting the minimum number of ADE cases required for statistical evaluations. Consequentially, key risk factors may have been underestimate, and potential safety signals for these drugs may have been underrepresented or missed in our findings. Moreover, there may be long-term skeletal risks associated with AEDs that were not captured in this study. For example, some AEDs are known to reduce bone mineral density and increase fracture risk, particularly with prolonged use. However, in this study, only two cases of osteoporosis-related ADEs were identified, which may reflect underreporting of the delayed onset of such outcomes in the spontaneous reporting system. Furthermore, the observational nature of this study’s design may result in unclear causality, as patient-specific factors, including unmeasured variables, may act as confounding factors that may influence the outcomes. Nonetheless, minimal bias from the ADE cases was expected in this study because the Korea Institute of Drug Safety and Risk Management (Ministry of Food and Drug Safety) performs in-depth investigations of ADE reports by reviewing patients’ medical charts, collecting scientific pharmacovigilance data from manufacturers, and consulting with healthcare professionals appointed by the institution. Despite these limitations, this study contributes valuable real-world evidence on the safety of AEDs, highlighting population-specific vulnerabilities, and accentuates the importance of integrating pharmacovigilance data into clinical decision-making to promote safe AED use. Furthermore, this study provides evidence for tailored guidance on the prevention of SAEs in elderly patients that can be applied in clinical practice.

## 4. Materials and Methods

### 4.1. Study Design and Data Collection

This cross-sectional study was conducted in accordance with the Strengthening the Reporting of Observational Studies in Epidemiology (STROBE) guidelines [29]. This study analyzed AED-related adverse event (AE) cases spontaneously reported to the KIDS KAERS DB, constructed by the Korean Institute of Drug Safety and Risk Management (KIDS, Ministry of Food and Drug Safety) from 1 January 2014, to 31 December 2023 [30,31,32]. The prespecified medications included 16 AEDs: carbamazepine, clonazepam divalproex, ethosuximide, gabapentin, lacosamide, lamotrigine, levetiracetam, oxcarbazepine, phenobarbital, phenytoin, pregabalin, primidone, topiramate, valproic acid, and zonisamide. The causality of drug-induced AEs was assessed by the World Health Organization–Uppsala Monitoring Centre (WHO–UMC) criteria, and all AEs with “certain”, “probable/likely”, and “possible” causality were included in the analysis. Any AEs with “unlikely”, “conditional/unclassified”, or “unassessable/unclassifiable” causality [33], and those with masked (MSK-coded) etiologic medications, were excluded from the analysis. MSK codes are assigned to medication products that are marketed by fewer than 2 pharmaceutical companies. Both monotherapy and polytherapy ADE cases were included in the analysis, with no exclusion applied based on concomitant medication use. All ADEs were reported in accordance with Medical Dictionary for Regulatory Activities (MedDRA) terminology and were further classified into system organ classes (SOCs). Serious adverse events (SAEs) were classified in accordance with the International Conference on Harmonization (ICH) E2D guidelines and included any ADEs involving death, life-threatening conditions, a persistent or significant disability or incapacity, hospitalization or prolonged existing hospitalizations, congenital abnormalities or birth defects, and other medically significant events [34]. The following data were extracted from KIDS KAERS DB: (1) patient demographic information, including sex and age, (2) medical histories and concurrent medication lists, (3) ADE information, including etiologic medications, causality assessment, occurrence date, resolution date, and seriousness, and (4) information on reporters. The protocol for utilizing KIDS KAERS DB was approved by KIDS (Ministry of Food and Drug Safety) (KIDS KAERS DB 2405A0010) and was reviewed and approved for exemption by the Kyung Hee University institutional board (IRB) (No. KHSIRB–24–418(EA), approved 14 August 2024) as the study involved de-identified, anonymized data.

### 4.2. Statistical Analysis

Descriptive statistics were calculated to summarize patient demographics and ADE types related to depression treatment. Age was expressed as the median and interquartile range (IQR) based on the Kolmogorov–Smirnov normality test. The disproportionality test was performed to determine the likelihood of SAEs being reported for ADEs with at least 4 reported cases of both nonserious ADEs and SAEs to ensure the validity and reliability of the results [31,32]. The disproportionality test was conducted, and the effect size was estimated as the reporting odds ratio (ROR) with corresponding 95% confidence interval (CIs) and Mantel–Haenszel-adjusted P-values for the following assessments: (1) the association between AEDs and SAEs, (2) the association of SOC-based ADEs with seriousness, (3) the association of SOC-based ADEs with seriousness for each AED agent, (4) the association of the type of concomitantly used medications with SAEs, and (5) the association of SOC-based ADEs and individual AED agents in both nonserious and serious ADE cases. The ROR was calculated based on Table 10.

A univariate logistic regression was performed to identify risk factors associated with AED-induced SAEs, and these factors included sex, age, causality, number of concurrently used medications, and types of AEDs or concomitantly used medications. A multiple logistic regression with the forward selection method was conducted to estimate the effect size of the predictors that were substantially associated with the seriousness of AED-induced ADEs based on the univariate logistic regression. The effect size was estimated using the odds ratio (OR) with a 95% CI. A sensitivity analysis of the AED-induced ADEs in elderly patients who were aged 60 years and older was carried out to determine the significance of ADEs in the elderly [35]. A Kaplan–Meier survival analysis and Cox’s proportional hazard modeling were performed to evaluate the differences in the duration of ADEs across SOC categories and in relation to the type of concomitant drug therapy used in elderly patients. Any ADEs with at least 100 reported cases were included in the analysis. Only ADE records with the reported occurrence and resolution date were analyzed, and the effect size was estimated using the hazard ratio (HR) with a 95% CI. All statistical analyses were conducted with SPSS Statistic 26.0 (IBM SPSS Statistics for Windows, Armonk, NY, USA) and R (version 4.3.1). Statistical significance was determined by any *p*-value < 0.05.

## 5. Conclusions

This study provides a comprehensive evaluation of AED-induced ADEs using a nationwide pharmacovigilance database. The study highlights significant variability in the frequency, severity, and duration of ADEs depending on the type of AEDs, patient demographics, and concomitant medication use. Elderly patients were more likely to report SAEs related to white cell and RES disorders and liver and biliary disorders. The multivariate analysis identified male sex, the use of immunosuppressants and chemotherapy agents, and specific AEDs as key predictors associated with an increased risk of SAEs. Among elderly patients, advanced aged and certain AEDs were important predictors of elevated SAE risk. The likelihood of SAEs being reported was lower with central and peripheral nervous system disorders and psychiatric disorders; however, patients experienced prolonged ADE durations related to these disorders. Interestingly, the concomitant use of analgesics, such as NSAIDs, and opioids was identified as a predictor associated with a lower SAE risk, despite insignificant durations of ADEs in the elderly. Conversely, concomitant AST was associated with prolonged ADE recovery. These findings strongly emphasize the importance of individualized AED selection along with cautious monitoring in elderly patients. Further controlled studies are warranted to explore the mechanism underlying prolonged ADE recovery to optimize epilepsy management across diverse patient populations.

## Figures and Tables

**Table 1:** Baseline demographic characteristics of patients.

Characteristics	No. of Cases (% Relative Frequency) or Median (IQR)
Age (years)	Median: 60 IQR: 24
0–9	827 (2.25%)
10–19	887 (2.41%)
20–29	1946 (5.29%)
30–39	2631 (7.15%)
40–49	4190 (11.38%)
50–59	7173 (19.49%)
60–69	8757 (23.79%)
70–79	7576 (20.58%)
80–89	2655 (7.21%)
90–99	167 (0.45%)
Sex	
Men	12,656 (34.38%)
Women	24,153 (65.62%)
Causality	
Certain	514 (1.40%)
Probable/Likely	7477 (20.31%)
Possible	28,818 (78.29%)
Seriousness	
Serious adverse events	1392 (3.78%)
Nonserious adverse events	35,417 (96.22%)
Reporting individuals	
Doctors	6503 (17.67%)
Pharmacists	18,232 (49.53%)
Nurses and other healthcare professionals	11,049 (30.02%)
General public	1025 (2.78%)
No. concomitantly used medications	
1	18,064 (49.07%)
2	4132 (11.23%)
3	4018 (10.92%)
4	3589 (9.75%)
≥5	7006 (19.03%)
Anticonvulsant types	
Gabapentin	11,062 (30.05%)
Divalproex	169 (0.46%)
Lamotrigine	1611 (4.38%)
Lacosamide	477 (1.30%)
Levetiracetam	2501 (6.79%)
Valproic Acid	404 (1.10%)
Ethosuximide	2 (0.01%)
Oxcarbazepine	1067 (2.90%)
Zonisamide	178 (0.48%)
Carbamazepine	2098 (5.70%)
Clonazepam	2540 (6.90%)
Topiramate	1576 (4.28%)
Phenobarbital	161 (0.44%)
Phenytoin	498 (1.35%)
Pregabalin	12,318 (33.46%)
Primidone	147 (0.40%)

Abbreviation: IQR—interquartile range.

**Table 2:** Association of anticonvulsant medication class with the seriousness of ADEs.

AEDs	Non-SAE (N = 35,417)	SAE (N = 1392)	Total (N = 36,809)	ROR (95%CI)	*p*-Value
Gabapentin	10,949 (30.91%)	113 (8.12%)	11,062 (30.05%)	0.20 (0.16–0.24)	<0.001
Divalproex	156 (0.44%)	13 (0.93%)	169 (0.46%)	2.13 (1.21–3.76)	0.009
Lamotrigine	1427 (4.03%)	184 (13.22%)	1611 (4.38%)	3.63 (3.08–4.27)	<0.001
Lacosamide	462 (1.30%)	15 (1.08%)	477 (1.30%)	0.82 (0.49–1.38)	0.464
Levetiracetam	2292 (6.47%)	209 (15.01%)	2501 (6.79%)	2.55 (2.19–2.98)	<0.001
Valproic Acid	328 (0.93%)	76 (5.46%)	404 (1.10%)	6.18 (4.79–7.98)	<0.001
Ethosuximide	2 (0.01%)	0 (0.00%)	2 (0.01%)	N/A	N/A
Oxcarbazepine	996 (2.81%)	71 (5.10%)	1067 (2.90%)	1.86 (1.45–2.38)	<0.001
Zonisamide	166 (0.47%)	12 (0.86%)	178 (0.48%)	1.85 (1.03–3.33)	0.041
Carbamazepine	1817 (5.13%)	281 (20.19%)	2098 (5.70%)	4.68 (4.07–5.38)	<0.001
Clonazepam	2483 (7.01%)	57 (4.09%)	2540 (6.90%)	0.57 (0.43–0.74)	<0.001
Topiramate	1521 (4.29%)	55 (3.95%)	1576 (4.28%)	0.92 (0.70–1.21)	0.535
Phenobarbital	140 (0.40%)	21 (1.51%)	161 (0.44%)	3.86 (2.43–6.13)	<0.001
Phenytoin	432 (1.22%)	66 (4.74%)	498 (1.35%)	4.03 (3.09–5.25)	<0.001
Pregabalin	12,101 (34.17%)	217 (15.59%)	12,318 (33.46%)	0.36 (0.31–0.41)	<0.001
Primidone	145 (0.41%)	2 (0.14%)	147 (0.40%)	0.35 (0.09–1.41)	0.141

**Table 3:** Association between system organ class (SOC)-based ADEs and seriousness caused by AEDs.

System Organ Class	Non-SAE (N = 35,417)	SAE (N = 1392)	Total (N = 36,809)	ROR (95% CI)	*p*-Value
Skin and appendages disorders	4243 (11.98%)	356 (25.57%)	4599 (12.49%)	2.53 (2.23–2.86)	<0.001
Musculoskeletal system disorders	191 (0.54%)	7 (0.50%)	198 (0.54%)	0.93 (0.44–1.99)	0.855
Collagen disorders	4 (0.01%)	1 (0.07%)	5 (0.01%)	6.37 (0.71–56.98)	0.098
Central and peripheral nervous system disorders	8119 (22.92%)	151 (10.85%)	8270 (22.47%)	0.41 (0.35–0.49)	<0.001
Vision disorders	284 (0.80%)	9 (0.65%)	293 (0.80%)	0.81 (0.41–1.57)	0.523
Hearing and vestibular disorders	39 (0.11%)	1 (0.07%)	40 (0.11%)	0.65 (0.09–4.75)	0.673
Special senses and other disorders	31 (0.09%)	0 (0.00%)	31 (0.08%)	N/A	N/A
Psychiatric disorders	4809 (13.58%)	43 (3.09%)	4852 (13.18%)	0.20 (0.15–0.28)	<0.001
Gastrointestinal system disorders	5199 (14.68%)	13 (0.93%)	5212 (14.16%)	0.06 (0.03–0.10)	<0.001
Liver and biliary system disorders	415 (1.17%)	82 (5.89%)	497 (1.35%)	5.28 (4.14–6.73)	<0.001
Metabolic and nutritional disorders	641 (1.81%)	21 (1.51%)	662 (1.80%)	0.83 (0.54–1.29)	0.407
Endocrine disorders	5 (0.01%)	3 (0.22%)	8 (0.02%)	15.30 (3.65–64.07)	<0.001
General cardiovascular disorders	115 (0.32%)	11 (0.79%)	126 (0.34%)	2.45 (1.31–4.55)	0.005
Heart rate and rhythm disorders	128 (0.36%)	7 (0.50%)	135 (0.37%)	1.39 (0.65–2.99)	0.394
Vascular (extracardiac) disorders	36 (0.10%)	1 (0.07%)	37 (0.10%)	0.71 (0.10–5.16)	0.732
Respiratory system disorders	7598 (21.45%)	378 (27.16%)	7976 (21.67%)	1.37 (1.21–1.54)	<0.001
Red blood cell disorders	23 (0.06%)	9 (0.65%)	32 (0.09%)	10.01 (4.63–21.68)	<0.001
White cell and RES disorders	229 (0.65%)	77 (5.53%)	306 (0.83%)	9.00 (6.91–11.72)	<0.001
Platelet, bleeding, and clotting disorders	44 (0.12%)	0 (0.00%)	44 (0.12%)	N/A	N/A
Urinary system disorders	615 (1.74%)	17 (1.22%)	632 (1.72%)	0.70 (0.43–1.14)	0.149
Reproductive disorders, male	12 (0.03%)	0 (0.00%)	12 (0.03%)	N/A	N/A
Reproductive disorders, female	42 (0.12%)	0 (0.00%)	42 (0.11%)	N/A	N/A
Neoplasms	5 (0.01%)	2 (0.14%)	7 (0.02%)	10.19 (1.98–52.57)	0.006
Body as a whole—general disorders	2517 (7.11%)	202 (14.51%)	2719 (7.39%)	2.22 (1.90–2.59)	<0.001
Application site disorders	5 (0.01%)	0 (0.00%)	5 (0.01%)	N/A	N/A
Resistance mechanism disorders	4 (0.01%)	0 (0.00%)	4 (0.01%)	N/A	N/A
Secondary terms—events	63 (0.18%)	1 (0.07%)	64 (0.17%)	0.40 (0.06–2.91)	0.368
Poison-specific terms	1 (0.00%)	0 (0.00%)	1 (0.00%)	N/A	N/A

Abbreviations: N/A—not applicable; RES—reticuloendothelial system; ROR—reporting odds ratio; SAE—serious adverse events

**Table 4:** Disproportionality analysis of SOC-based ADEs by individual AEDs.

AEDs	Non-SAEs		SAEs	
	ROR (95% CI)	*p*-Value	ROR (95% CI)	*p*-Value
Skin and Appendages Disorder				
Gabapentin	0.31 (0.29–0.34)	<0.001	0.53 (0.31–0.89)	0.016
Divalproex	1.83 (1.23–2.71)	0.003	N/A	N/A
Lamotrigine	12.46 (11.15–13.92)	<0.001	3.30 (2.40–4.55)	<0.001
Lacosamide	1.10 (0.84–1.44)	0.502	N/A	N/A
Levetiracetam	2.42 (2.19–2.68)	<0.001	0.40 (0.27–0.61)	<0.001
Valproic acids	2.48 (1.93–3.19)	<0.001	0.70 (0.39–1.25)	0.232
Oxcarbazepine	6.80 (5.98–7.74)	<0.001	1.24 (0.73–2.09)	0.428
Zonisamide	1.12 (0.72–1.76)	0.613	2.09 (0.66–6.64)	0.209
Carbamazepine	5.10 (4.61–5.64)	<0.001	2.17 (1.64–2.87)	<0.001
Clonazepam	0.43 (0.36–0.51)	<0.001	0.53 (0.26–1.10)	0.089
Topiramate	0.66 (0.55–0.79)	<0.001	0.64 (0.32–1.28)	0.203
Phenobarbital	5.41 (3.86–7.58)	<0.001	0.68 (0.23–20.38)	0.492
Phenytoin	5.18 (4.26–6.29)	<0.001	2.73 (1.66–4.50)	<0.001
Pregabalin	0.2 (0.19–0.23)	<0.001	0.20 (0.12–0.33)	<0.001
Primidone	0.26 (0.11–0.64)	0.003	N/A	N/A
Musculoskeletal Disorder				
Gabapentin	0.97 (0.72–1.33)	0.869	N/A	N/A
Divalproex	1.19 (0.17–8.55)	0.862	4.32 (2.24–8.36)	<0.001
Lacosamide	2.05 (0.84–5.00)	0.116	N/A	N/A
Levetiracetam	0.97 (0.54–1.74)	0.915	N/A	N/A
Oxcarbazepine	0.74 (0.27–1.99)	0.549	N/A	N/A
Carbamazepine	1.02 (0.54–1.94)	0.947	N/A	N/A
Clonazepam	1.47 (0.91–2.36)	0.113	N/A	N/A
Topiramate	2.18 (1.54–4.00)	<0.001	N/A	N/A
Pregabalin	0.80 (0.58–1.09)	0.157	N/A	N/A
Central and Peripheral Nervous System Disorder				
Gabapentin	1.20 (1.14–1.27)	<0.001	1.63 (0.96–2.79)	0.072
Divalproex	0.55 (0.35–0.87)	0.01	N/A	N/A
Lamotrigine	0.14 (0.11–0.18)	<0.001	N/A	N/A
Lacosamide	1.35 (1.10–1.66)	0.004	N/A	N/A
Levetiracetam	0.39 (0.34–0.45)	<0.001	0.80 (0.48–1.32)	0.377
Valproic acids	0.63 (0.47–0.85)	0.002	4.96 (2.98–8.24)	<0.001
Oxcarbazepine	0.45 (0.37–0.54)	<0.001	0.61 (0.24–1.54)	0.295
Zonisamide	0.54 (0.35–0.84)	0.006	N/A	N/A
Carbamazepine	0.59 (0.52–0.67)	<0.001	0.25 (0.13–0.49)	<0.001
Clonazepam	0.55 (0.49–0.62)	<0.001	2.85 (1.52–5.34)	0.001
Topiramate	1.03 (0.91–1.16)	0.693	1.42 (0.66–3.07)	0.371
Phenobarbital	0.15 (0.07–0.34)	<0.001	N/A	N/A
Phenytoin	0.86 (0.68–1.09)	0.204	N/A	N/A
Pregabalin	1.71 (1.63–1.80)	<0.001	2.64 (1.80–3.88)	<0.001
Primidone	1.93 (1.37–2.71)	<0.001	N/A	N/A
Vision Disorders				
Gabapentin	0.50 (0.37–0.67)	<0.001	N/A	N/A
Divalproex	4.15 (1.69–10.20)	0.002	N/A	N/A
Lamotrigine	0.87 (0.46–1.64)	0.662	N/A	N/A
Lacosamide	6.62 (4.24–10.33)	<0.001	N/A	N/A
Levetiracetam	0.86 (0.52–1.43)	0.565	N/A	N/A
Valproic acids	2.74 (1.28–5.85)	0.009	N/A	N/A
Oxcarbazepine	2.80 (1.79–4.38)	<0.001	N/A	N/A
Carbamazepine	0.82 (0.46–1.46)	0.488	N/A	N/A
Clonazepam	0.69 (0.4–1.18)	0.17	N/A	N/A
Topiramate	2.37 (1.59–3.53)	<0.001	54.45 (13.23–224.12)	<0.001
Pregabalin	0.91 (0.71–1.17)	0.449	N/A	N/A
Psychiatric Disorders				
Gabapentin	1.40 (1.31–1.49)	<0.001	N/A	N/A
Divalproex	0.88 (0.55–1.43)	0.609	N/A	N/A
Lamotrigine	0.29 (0.22–0.37)	<0.001	N/A	N/A
Lacosamide	0.93 (0.71–1.23)	0.61	N/A	N/A
Levetiracetam	1.06 (0.94–1.20)	0.32	1.31 (0.60–2.86)	0.504
Valproic acids	1.04 (0.76–1.42)	0.813	N/A	N/A
Oxcarbazepine	0.37 (0.28–0.48)	<0.001	N/A	N/A
Zonisamide	1.58 (1.08–2.32)	0.019	N/A	N/A
Carbamazepine	0.45 (0.38–0.54)	<0.001	N/A	N/A
Clonazepam	2.40 (2.18–2.64)	<0.001	9.74 (4.62–20.52)	<0.001
Topiramate	1.41 (1.21–1.61)	<0.001	7.50 (3.40–16.54)	<0.001
Phenobarbital	0.76 (0.45–1.31)	0.323	N/A	N/A
Phenytoin	0.32 (0.21–0.50)	<0.001	N/A	N/A
Pregabalin	0.70 (0.65–0.74)	<0.001	1.25 (0.57–2.73)	0.58
Primidone	1.02 (0.64–1.63)	0.94	N/A	N/A
Gastrointestinal Disorders				
Gabapentin	1.70 (1.60–1.80)	<0.001	N/A	N/A
Divalproex	1.01 (0.65–1.57)	0.982	N/A	N/A
Lamotrigine	0.20 (0.15–0.27)	<0.001	N/A	N/A
Lacosamide	0.39 (0.27–0.56)	<0.001	N/A	N/A
Levetiracetam	0.93 (0.83–1.05)	0.26	N/A	N/A
Valproic acids	0.32 (0.19–0.51)	<0.001	N/A	N/A
Oxcarbazepine	0.37 (0.29–0.48)	<0.001	N/A	N/A
Zonisamide	0.62 (0.37–1.04)	0.068	N/A	N/A
Carbamazepine	0.42 (0.35–0.51)	<0.001	N/A	N/A
Clonazepam	1.00 (0.89–1.12)	0.977	N/A	N/A
Topiramate	0.69 (0.59–0.82)	<0.001	N/A	N/A
Phenobarbital	0.45 (0.23–0.85)	0.014	N/A	N/A
Phenytoin	0.28 (0.18–0.44)	<0.001	N/A	N/A
Pregabalin	1.07 (1.01–1.14)	0.03	N/A	N/A
Primidone	0.82 (0.5–1.35)	0.441	N/A	N/A
Liver and Biliary Disorders				
Gabapentin	0.17 (0.12–0.25)	<0.001	1.06 (0.48–2.36)	0.886
Lamotrigine	1.47 (0.97–2.23)	0.069	0.32 (0.12–0.89)	0.029
Lacosamide	0.92 (0.38–2.24)	0.857	N/A	N/A
Levetiracetam	12.82 (10.51–15.63)	<0.001	9.52 (5.96–15.19)	<0.001
Valproic acids	6.02 (3.83–9.48)	<0.001	N/A	N/A
Oxcarbazepine	1.48 (0.91–2.42)	0.114	N/A	N/A
Zonisamide	3.76 (1.75–8.07)	<0.001	N/A	N/A
Carbamazepine	1.29 (0.87–1.91)	0.203	0.24 (0.10–0.61)	0.002
Clonazepam	0.92 (0.62–1.37)	0.685	N/A	N/A
Topiramate	1.17 (0.73–1.89)	0.512	N/A	N/A
Phenobarbital	2.50 (0.92–6.78)	0.073	N/A	N/A
Phenytoin	4.96 (3.22–7.64)	<0.001	2.33 (1.08–5.07)	0.032
Pregabalin	0.12 (0.08–0.18)	<0.001	N/A	N/A
Metabolic disorders				
Gabapentin	0.68 (0.57–0.82)	<0.001	N/A	N/A
Divalproex	1.80 (0.74–4.41)	0.197	N/A	N/A
Lamotrigine	0.57 (0.34–0.95)	0.03	N/A	N/A
Lacosamide	0.71 (0.32–1.60)	0.409	N/A	N/A
Levetiracetam	1.98 (1.55–2.52)	<0.001	N/A	N/A
Valproic acids	4.62 (3.05–7.00)	<0.001	N/A	N/A
Oxcarbazepine	3.10 (2.31–4.15)	<0.001	12.78 (5.11–31.94)	<0.001
Zonisamide	2.40 (1.12–5.15)	0.024	N/A	N/A
Carbamazepine	1.17 (0.84–1.63)	0.356	0.93 (0.31–2.78)	0.896
Clonazepam	1.03 (0.76–1.39)	0.868	N/A	N/A
Topiramate	2.58 (1.99–3.36)	<0.001	N/A	N/A
Pregabalin	0.60 (0.50–0.72)	<0.001	N/A	N/A
Cardiovascular Disorders				
Gabapentin	1.34 (0.92–1.95)	0.134	N/A	N/A
Levetiracetam	N/A	N/A	3.28 (0.95–11.30)	0.06
Carbamazepine	0.67 (0.25–1.81)	0.424	N/A	N/A
Clonazepam	1.26 (0.66–2.42)	0.479	N/A	N/A
Topiramate	2.13 (1.11–4.08)	0.023	N/A	N/A
Pregabalin	0.59 (0.38–0.91)	0.017	N/A	N/A
Heart Rate and Rhythm Disorders				
Gabapentin	0.98 (0.67–1.43)	0.913	N/A	N/A
Levetiracetam	1.36 (0.73–2.53)	0.33	N/A	N/A
Lacosamide	2.45 (0.90–6.67)	0.079	N/A	N/A
Carbamazepine	0.75 (0.31–1.84)	0.531	N/A	N/A
Clonazepam	1.13 (0.59–2.15)	0.722	N/A	N/A
Topiramate	4.55 (2.89–7.16)	<0.001	N/A	N/A
Pregabalin	0.54 (0.36–0.82)	0.003	N/A	N/A
Vascular (Extracardiac) Disorders				
Gabapentin	0.75 (0.35–1.58)	0.444	N/A	N/A
Oxcarbazepine	4.33 (1.53–12.28)	0.006	N/A	N/A
Pregabalin	0.74 (0.36–1.54)	0.42	N/A	N/A
Respiratory Disorders				
Gabapentin	0.76 (0.71–0.80)	<0.001	1.17 (0.77–1.78)	0.465
Divalproex	0.80 (0.53–1.21)	0.286	1.69 (0.55–5.19)	0.362
Lamotrigine	0.83 (0.72–0.95)	0.007	0.13 (0.09–0.18)	<0.001
Lacosamide	1.13 (0.91–1.41)	0.26	7.57 (2.40–23.92)	<0.001
Levetiracetam	0.54 (0.48–0.61)	<0.001	0.80 (0.56–1.12)	0.192
Valproic acids	0.52 (0.38–0.72)	<0.001	0.44 (0.23–0.84)	0.013
Oxcarbazepine	0.55 (0.45–0.66)	<0.001	0.84 (0.48–1.46)	0.533
Zonisamide	0.98 (0.69–1.42)	0.908	N/A	N/A
Carbamazepine	0.77 (0.68–0.88)	<0.001	0.85 (0.63–1.14)	0.273
Clonazepam	1.22 (1.11–1.34)	<0.001	0.56 (0.28–1.12)	0.1
Topiramate	0.81 (0.70–0.92)	0.001	0.51 (0.25–1.06)	0.071
Phenobarbital	0.01 (0.28–0.79)	0.471	2.48 (1.04–5.88)	0.04
Phenytoin	0.34 (0.24–0.48)	<0.001	0.16 (0.06–0.46)	<0.001
Pregabalin	1.74 (1.65–1.83)	<0.001	2.89 (2.14–3.90)	<0.001
Primidone	1.40 (0.97–2.01)	0.073	N/A	N/A
Red Blood Cell Disorders				
Gabapentin	0.62 (0.23–1.67)	0.45	N/A	N/A
Levetiracetam	3.05 (1.04–8.96)	0.043	N/A	N/A
Oxcarbazepine	N/A	N/A	15.71 (4.13–59.86)	<0.001
Carbamazepine	3.90 (1.33–11.47)	0.013	N/A	N/A
White Cell and RES disorders				
Gabapentin	0.62 (0.23–1.67)	0.345	N/A	N/A
Divalproex	4.10 (1.51–11.16)	0.006	N/A	N/A
Lamotrigine	0.64 (0.28–1.44)	0.281	0.35 (0.13–0.96)	0.041
Levetiracetam	9.52 (7.27–12.46)	<0.001	3.81 (2.34–6.20)	<0.001
Valproic acids	3.94 (1.93–8.05)	<0.001	1.22 (0.48–3.11)	0.682
Oxcarbazepine	2.62 (1.57–4.38)	<0.001	3.07 (1.51–6.26)	0.002
Carbamazepine	2.82 (1.91–4.15)	<0.001	0.44 (0.21–0.93)	0.032
clonazepam	0.48 (0.24–0.97)	0.041		
Topiramate	1.23 (0.65–2.34)	0.521	N/A	N/A
Phenobarbital	5.52 (2.02–15.07)	<0.001	N/A	N/A
Phenytoin	8.20 (4.93–13.61)	<0.001	N/A	N/A
Pregabalin	0.16 (0.10–0.28)	<0.001	N/A	N/A
Urinary System Disorders				
Gabapentin	1.87 (1.59–2.19)	<0.001	N/A	N/A
Divalproex	1.49 (0.55–4.04)	0.431	N/A	N/A
Lamotrigine	0.15 (0.06–0.41)	<0.001	N/A	N/A
Levetiracetam	0.56 (0.37–0.85)	0.006	N/A	N/A
Oxcarbazepine	0.34 (0.15–0.75)	0.008	N/A	N/A
Zonisamide	1.76 (0.72–4.31)	0.213	N/A	N/A
Carbamazepine	0.55 (0.35–0.89)	0.014	N/A	N/A
Clonazepam	1.02 (0.75–1.39)	0.888	N/A	N/A
Topiramate	0.63 (0.60–1.36)	0.903	N/A	N/A
Phenytoin	N/A	N/A	8.98 (3.07–26.28)	<0.001
Pregabalin	0.49 (0.79–1.12)	0.941	2.29 (0.80–6.56)	0.125
Body as a Whole Disorders				
Gabapentin	1.32 (1.21–1.44)	<0.001	1.30 (0.78–2.15)	0.317
Divalproex	0.90 (0.47–1.70)	0.734	N/A	N/A
Lamotrigine	0.87 (0.70–1.08)	0.192	1.40 (0.94–2.11)	0.102
Levetiracetam	0.72 (0.59–0.86)	<0.001	0.41 (0.24–0.71)	0.002
Valproic acids	0.94 (0.61–1.45)	0.777	0.58 (0.26–1.29)	0.182
Oxcarbazepine	0.60 (0.44–0.81)	<0.001	0.03 (0.01–0.10)	<0.001
Zonisamide	0.07 (0.04–0.14)	<0.001	2.99 (0.89–10.01)	0.076
Carbamazepine	0.92 (0.76–1.11)	0.392	2.53 (1.83–3.50)	<0.001
Clonazepam	0.85 (0.72–1.01)	0.058	0.82 (0.37–1.83)	0.626
Topiramate	0.93 (0.75–1.14)	0.469	N/A	N/A
Phenobarbital	1.46 (0.84–2.53)	0.184	1.39 (0.46–4.19)	0.554
Phenytoin	0.70 (0.45–1.08)	0.103	0.91 (0.44–1.87)	0.797
Pregabalin	1.02 (0.93–1.11)	0.727	0.66 (0.42–1.05)	0.077
Primidone	0.66 (0.31–1.42)	0.288	N/A	N/A

**Table 5:** Association of type of concomitantly used medication class with seriousness of ADEs.

Types of Concomitantly Used Medication	Non-SAE (N = 35,457)	SAE (N = 1392)	Total (N = 36,809)	ROR (95%CI)	*p*-Value
Acid-suppression therapy	3671 (14.49%)	22 (6.9%)	3693 (14.4%)	0.14 (0.09–0.21)	<0.001
H2RA	2671 (10.54%)	9 (2.82%)	2680 (10.45%)	0.08 (0.04–0.15)	<0.001
PPI	972 (3.84%)	12 (3.76%)	984 (3.84%)	0.31 (0.17–0.55)	<0.001
Rabeprazole	322 (1.27%)	9 (2.82%)	331 (1.25%)	0.71 (0.37–1.38)	0.311
H2RA and PPI combinations	28 (0.11%)	1 (0.31%)	29 (0.11%)	0.91 (0.12–6.68)	0.925
Acetaminophen	4875 (19.24%)	45 (14.11%)	4920 (19.18%)	0.21 (0.16–0.28)	<0.001
NSAIDs	6064 (23.94%)	71 (22.26%)	6135 (23.92%)	0.26 (0.21–0.33)	<0.001
Loxoprofen	896 (3.54%)	5 (1.57%)	901 (3.51%)	0.14 (0.06–0.34)	<0.001
Celecoxib	1314 (5.19%)	24 (7.52%)	1338 (5.22%)	0.46 (0.30–0.68)	<0.001
Aceclofenac	1522 (6.01%)	14 (4.39%)	1536 (5.99%)	0.23 (0.13–0.38)	<0.001
Ibuprofen	172 (0.68%)	6 (1.88%)	178 (0.69%)	0.89 (0.39–2.01)	0.773
Multiple NSAID combinations	64 (0.25%)	5(1.57%)	69 (0.27%)	1.99 (0.80–4.96)	0.139
Opioids	4889 (19.30%)	37 (11.60%)	4926 (19.20%)	0.17 (0.12–0.24)	<0.001
Tramadol	4525 (17.86%)	30 (9.4%)	4555 (17.76%)	0.15 (0.11–0.22)	<0.001
Benzodiazepines	1289 (5.09%)	43 (13.48%)	1332 (5.19%)	0.84 (0.62–1.15)	0.281
Zolpidem	293 (1.16%)	5(1.57%)	298 (1.16%)	0.43 (0.18–1.05)	0.063
Antidepressants	2182 (8.61%)	30 (9.4%)	2212 (8.62%)	0.34 (0.23–0.48)	<0.001
Amitriptyline	776 (3.06%)	9 (2.82%)	785 (3.06%)	0.29 (0.15–0.56)	<0.001
Duloxetine	370 (1.46%)	5 (1.57%)	375 (0.50%)	0.34 (0.14–0.83)	0.017
Sedatives	122 (0.48%)	6(1.88%)	128 (0.5%)	1.25 (0.55–2.85)	0.591
Dementia treatment	434 (1.71%)	11 (3.45%)	445 (1.73%)	0.64 (0.35–1.17)	0.148
Immunosuppressants	75 (0.30%)	5 (1.57%)	80 (0.31%)	1.70 (0.69–4.21)	0.252
Chemotherapy	132 (0.52%)	10 (3.13%)	142 (0.55%)	1.93 (1.02–3.69)	0.045
Antihyperglycemic Treatments	454 (1.79%)	7 (2.19%)	461 (1.8%)	0.39 (0.18–0.82)	0.013

Abbreviations: H2RA—histamine-2 receptor antagonists; PPI—proton pump inhibitor.

**Table 6:** System organ class (SOC)-based ADEs caused by anticonvulsants in elderly patients.

System Organ Class	Non-SAE (N = 18,566)	SAE (N = 589)	Total (N = 19,155)	ROR	*p*-Value
Skin and appendages disorders	1479 (7.97%)	114 (19.35%)	1593 (8.32%)	2.77 (2.25–3.43)	<0.001
Musculoskeletal system disorders	103 (0.55%)	2 (0.34%)	105 (0.55%)	0.61 (0.15–2.48)	0.491
Collagen disorders	1 (0.01%)	0 (0.00%)	1 (0.01%)	N/A	N/A
Central and peripheral nervous system disorders	4562 (24.57%)	75 (12.73%)	4637 (24.21%)	0.45 (0.35–0.57)	<0.001
Vision disorders	119 (0.64%)	1 (0.17%)	120 (0.63%)	0.26 (0.04–1.89)	0.185
Hearing and vestibular disorders	7 (0.04%)	1 (0.17%)	8 (0.04%)	4.51 (0.55–36.71)	0.159
Special senses and other disorders	17 (0.09%)	0 (0.00%)	17 (0.09%)	N/A	N/A
Psychiatric disorders	2342 (12.61%)	19 (3.23%)	2361 (12.33%)	0.23 (0.15–0.37)	<0.001
Gastrointestinal system disorders	2886 (15.54%)	3 (0.51%)	2889 (15.08%)	0.03 (0.01–0.09)	<0.001
Liver and biliary system disorders	169 (0.91%)	32 (5.43%)	201 (1.05%)	6.25 (4.25–9.21)	<0.001
Metabolic and nutritional disorders	269 (1.45%)	14 (2.38%)	283 (1.48%)	1.66 (0.96–2.85)	0.069
Endocrine disorders	0 (0.00%)	3 (0.51%)	3 (0.02%)	N/A	N/A
General cardiovascular disorders	62 (0.33%)	6 (1.02%)	68 (0.35%)	3.07 (1.32–7.13)	0.009
Heart rate and rhythm disorders	55 (0.3%)	4 (0.68%)	59 (0.31%)	2.30 (0.83–6.37)	0.109
Vascular (extracardiac) disorders	21 (0.11%)	0 (0.00%)	21 (0.11%)	N/A	N/A
Respiratory system disorders	4525 (24.37%)	191 (32.43%)	4716 (24.62%)	1.49 (1.25–1.78)	<0.001
Red blood cell disorders	11 (0.06%)	0 (0.00%)	11 (0.06%)	N/A	N/A
White cell and RES disorders	92 (0.50%)	30 (5.09%)	122 (0.64%)	10.78 (7.08–16.41)	<0.001
Platelet, bleeding, and clotting disorders	29 (0.16%)	0 (0.00%)	29 (0.15%)	N/A	N/A
Urinary system disorders	415 (2.24%)	11 (1.87%)	426 (2.22%)	0.83 (0.46–1.52)	0.552
Reproductive disorders, male	1 (0.01%)	0 (0.00%)	1 (0.01%)	N/A	N/A
Reproductive disorders, female	2 (0.01%)	0 (0.00%)	2 (0.01%)	N/A	N/A
Body as a whole—general disorders	1364 (7.35%)	83 (14.09%)	1447 (7.55%)	2.07 (1.63–2.63)	<0.001
Application site disorders	1 (0.01%)	0 (0.00%)	1 (0.01%)	N/A	N/A
Resistance mechanism disorders	2 (0.01%)	0 (0.00%)	2 (0.01%)	N/A	N/A
Secondary terms—events	32 (0.17%)	0 (0.00%)	32 (0.17%)	N/A	N/A

Abbreviations: N/A—not applicable; RES—reticuloendothelial system; ROR—reporting odds ratio; SAE—serious adverse events

**Table 7:** Association of types of concomitantly used medication class with seriousness of ADEs in elderly patients.

Types of Concomitantly Used Medication	Non-SAE (N = 18,566)	SAE (N = 589)	Total (N = 19,155)	ROR (95%CI)	*p*-Value
Acid-suppression therapy	2050 (14.23%)	9 (6.21%)	2059 (14.15%)	0.13 (0.07–0.24)	<0.001
Acetaminophen	2990 (20.75%)	24 (16.55%)	3014 (20.71%)	0.22 (0.15–0.33)	<0.001
NSAIDs	3482 (24.16%)	40 (27.59%)	3522 (24.20%)	0.32 (0.23–0.44)	<0.001
Celecoxib	975 (6.77%)	11 (7.59%)	986 (6.77%)	0.34 (0.19–0.63)	<0.001
Aceclofenac	815 (5.66%)	7 (4.83%)	822 (5.65%)	0.26 (0.12–0.55)	<0.001
Opioids	3056 (21.21%)	22 (15.17%)	3078 (21.15%)	0.20 (0.13–0.30)	<0.001
Tramadol	2829 (19.63%)	18 (12.41%)	2847 (19.56%)	0.18 (0.11–0.28)	<0.001
Benzodiazepines	443 (3.07%)	12 (8.28%)	455 (3.13%)	0.85 (0.48–1.52)	0.585
Antidepressants	1106 (7.67%)	9 (6.21%)	1115 (7.66%)	0.25 (0.13–0.48)	<0.001
Antipsychotics	189 (1.31%)	6 (4.14%)	195 (1.34%)	1.00 (0.44–2.27)	0.999
Dementia treatment	361 (2.51%)	9 (6.21%)	370 (2.54%)	0.78 (0.40–1.52)	0.471
Antihyperglycemic Treatments	330 (2.29%)	5 (3.45%)	335 (2.30%)	0.47 (0.20–1.15)	0.098

**Table 8:** Univariate and multivariate analyses for the identification of predictors of SAEs in the general population.

Predictors	Number of Cases (%)		Univariate Analysis		Multivariate Analysis	
	Nonserious AEs (N = 35,417)	SAEs (N = 1392)	OR (95% CI)	*p*-Value	OR (95% CI)	*p*-Value
Sex						
Male	12,008 (33.90%)	648 (46.55%)	1.70 (1.53–1.89)	<0.001	1.36 (1.21–1.52)	<0.001
Female	23,409 (66.10%)	744 (53.45%)	Reference		Reference	
Age						
0–9	772 (2.18%)	55 (3.95%)	0.98 (0.96–1.00)	0.039	N/A	N/A
10–19	792 (2.24%)	95 (6.82%)				
20–29	1818 (5.13%)	128 (9.20%)				
30–39	2493 (7.04%)	138 (9.91%)				
40–49	4036 (11.40%)	154 (11.06%)				
50–59	6940 (19.60%)	233 (16.74%)				
60–69	8501 (24.00%)	256 (18.39%)				
70–79	7344 (20.74%)	232 (16.67%)				
80–89	2561 (7.23%)	94 (6.75%)				
90–99	160 (0.45%)	7 (0.50%)				
Causality						
Certain	464 (1.31%)	50 (3.59%)	Reference	<0.001	reference	<0.001
Probable/likely	7003 (19.77%)	474 (34.05%)	0.62 (0.44–0.85)	0.004	0.63 (0.46–0.87)	0.005
Possible	27,950 (78.92%)	868 (62.36%)	0.27 (0.20–0.37)	<0.001	0.47 (0.34–0.64)	<0.001
Number of concurrently used medications						
1	17,166 (48.47%)	898 (64.51%)	0.67 (0.64–0.71)	<0.001	0.89 (0.84–0.94)	<0.001
2	3901 (11.01%)	231 (16.59%)				
3	3897 (11.00%)	121 (8.69%)				
4	3535 (9.98%)	54 (3.88%)				
≥5	6918 (19.35%)	88 (6.32%)				
Types of concomitantly used medication						
Acid-suppressive therapy	3671 (10.37%)	22 (1.58%)	0.17 (0.11–0.26)	<0.001	0.38 (0.24–0.60)	<0.001
Anesthetics	15 (0.04%)	0 (0.00%)	N/A	N/A	N/A	N/A
Steroids	11 (0.03%)	0 (0.00%)	N/A	N/A	N/A	N/A
Acetaminophen	4875 (13.76%)	45 (3.23%)	N/A	N/A	N/A	N/A
NSAIDs	6064 (17.12%)	71 (5.10%)	N/A	N/A	N/A	N/A
Opioids	4889 (13.80%)	37 (2.66%)	0.20 (0.14–0.28)	<0.001	0.62 (0.43–0.89)	0.010
Benzodiazepines	1289 (3.64%)	43 (3.09%)	N/A	N/A	N/A	N/A
Neurological disorders	217 (0.61)	3 (0.22%)	N/A	N/A	N/A	N.A
Zolpidem	293 (0.83%)	5 (0.36%)	N/A	N/A	N/A	N/A
Antidepressants	2182 (6.16%)	30 (2.16%)	0.35 (0.24–0.50)	<001	0.57 (0.39–0.84)	0.005
Sedatives	122 (0.34%)	6 (0.43%)	N/A	N/A	N/A	N/A
Antipsychotics	611 (1.73%)	24 (1.72$)	N/A	N/A	N/A	N/A
Dementia treatment	434 (1.23%)	11 (0.79%)	N/A	N/A	N/A	N/A
Immunosuppressants	75 (0.21%)	5 (0.36%)	2.62 (1.04–6.64)	0.042	3.68 (1.39–9.74)	0.009
Chemotherapy	132 (0.37%)	10 (0.72%)	2.51 (1.30–4.83)	0.006	2.22 (1.11–4.45)	0.024
Diabetic medications	454 (1.28%)	7 (0.50%)	0.42 (0.20–0.89)	0.024	N/A	N/A
Anticonvulsant Types						
Gabapentin	10,949 (30.91%)	113 (8.12%)	0.43 (0.32–0.58)	<0.001	0.42 (0.32–0.54)	<0.001
Divalproex	156 (0.44%)	13 (0.93%)	3.48 (1.89–6.42)	<0.001	3.06 (1.69–5.57)	<0.001
Lamotrigine	1427 (4.03%)	184 (13.22%)	5.39 (4.08–7.11)	<0.001	3.43 (2.70–4.35)	<0.001
Lacosamide	462 (1.30%)	15 (1.08%)	N/A	N/A	N/A	N/A
Levetiracetam	2292 (6.47%)	209 (15.01%)	3.81 (2.91–4.99)	<0.001	2.61 (2.08–3.28)	<0.001
Valproic acid	328 (0.93%)	76 (5.46%)	9.68 (6.89–13.60)	<0.001	7.88 (5.77–10.77)	<0.001
Ethosuximide	2 (0.01%)	0 (0.00%)	N/A	NA	N/A	N/A
Oxcarbazepine	996 (2.81%)	71 (5.10%)	2.98 (2.13–1.16)	<0.001	1.98 (1.46–2.67)	<0.001
Zonisamide	166 (0.47%)	12 (0.86%)	3.02 (1.61–5.67)	<0.001	2.15 (1.16–3.98)	0.014
Carbamazepine	1817 (5.13%)	281 (20.19%)	6.46 (4.97–8.40)	<0.001	4.47 (3.59–5.57)	<0.001
Clonazepam	2483 (7.01%)	57 (4.09%)	N/A	N/A	N/A	N/A
Topiramate	1521 (4.29%)	55 (3.95%)	1.51 (1.06–2.15)	0.022	N/A	N/A
Phenobarbital	140 (0.40%)	21 (1.51%)	6.27 (3.75–10.47)	<0.001	4.49 (2.73–7.36)	<0.001
Phenytoin	432 (1.22%)	66 (4.74%)	6.38 (4.51–9.03)	<0.001	4.02 (2.92–5.52)	<0.001
Pregabalin	12,101 (34.17%)	217 (15.59%)	0.75 (0.75–0.98)	0.034	0.63 (0.50–0.79)	<0.001
Primidone	145 (0.41%)	2 (0.14%)	N/A	N/A	N/A	N/A

**Table 9:** Univariate and multivariate analyses for the identification of predictors for SAEs in elderly patients.

Predictors	Number of Cases (%)		Univariate Analysis		Multivariate Analysis	
	Nonserious AEs (N = 18,566)	SAEs (N = 589)	OR (95% CI)	*p*-Value	OR (95% CI)	*p*-Value
Sex						
Male	6090 (32.8%)	306 (51.95%)	2.22 (1.88–2.61)	<0.001	1.91 (1.62–2.27)	<0.001
Female	12,476 (67.2%)	283 (48.05%)	reference		Reference	
Age						
60–69	8501 (45.79%)	256 (43.46%)	N/A	N/A	1.17 (1.04–1.31)	0.007
70–79	7344 (39.56%)	232 (39.39%)				
80–89	2561 (13.79%)	94 (15.96%)				
90–99	160 (0.86%)	7 (1.19%)				
Causality						
Certain	252 (1.36%)	18 (3.06%)	Reference	<0.001	Reference	0.002
Probable/likely	3397 (1.8.30%)	175 (29.71%)	0.72 (0.44–1.19)	0.202	0.76 (0.45–1.28)	0.298
Possible	14,917 (80.35%)	396 (67.23%)	0.37 (0.23–0.61)	<0.001	0.52 (0.34–0.94)	0.027
Number of concurrently used medications						
1	8735 (47.05%)	385 (65.37%)	0.72 (0.67–0.76)	<0.001	N/A	N/A
2	2100 (11.31%)	86 (14.6%)				
3	2080 (11.20%)	46 (7.81%)				
4	1892 (10.19%)	28 (4.75%)				
≥5	3759 (20.25%)	44(7.47%)				
Types of concomitantly used medication						
Acid Suppressive Therapy	2050 (11.04%)	9 (1.53%)	0.21 (0.11–0.42)	<0.001	0.23 (0.12–0.46)	<0.001
NSAIDs	3482 (18.75%)	40 (6.79%)	0.50 (0.36–0.70)	<0.001	N/A	N/A
Opioids	3056 (16.46%)	22 (3.74%)	0.26 (0.17–0.40)	<0.001	0.43 (0.28–0.67)	<0.001
Antidepressants	1106 (5.96%)	9 (1.53%)	0.25 (0.13–0.48)	<0.001	0.34 (0.17–0.66)	0.002
Neurological disorders	106 (0.57%)	1 (0.17%)	N/A	N/A	N/A	N/A
Anesthetics	12 (0.06%)	0 (0.00%)	N/A	N/A	N/A	N/A
Steroids	9 (0.05%)	0 (0.00%)	N/A	N/A	N/A	N/A
Acetaminophen	2990 (16.10%)	24 (4.07%)	N/A	N/A	N/A	N/A
Benzodiazepines	443 (2.39%)	12 (2.04%)	N/A	N/A	N/A	N/A
Zolpidem	136 (0.73%)	4 (0.68%)	N/A	N/A	N/A	N/A
Antipsychotics	189 (1.02%)	6 (1.02%)	N/A	N/A	N/A	N/A
Sedatives	45 (0.24%)	0 (0.00%)	N/A	N/A	N/A	N/A
Dementia treatment	361 (1.94%)	9 (1.53%)	N/A	N/A	N/A	N/A
Immunosuppressants	31 (0.17%)	1 (0.17%)	N/A	N/A	N/A	N/A
Chemotherapy	65 (0.35%)	3 (0.51%)	N/A	N/A	N/A	N/A
Diabetic medications	330 (1.78%)	5 (0.85%)	N/A	N/A	N/A	N/A
Anticonvulsant Types						
Gabapentin	6258 (33.71%)	65 (11.04%)	0.20 (0.14–0.30)	<001	0.26 (0.18–0.39)	<001
Divalproex	27 (0.15%)	1 (0.17%)	N/A	N/A	N/A	N/A
Lamotrigine	285 (1.54%)	40 (6.79%)	2.73 (1.75–4.28)	<001	2.26 (1.43–3.56)	<001
Lacosamide	125 (0.67%)	9 (1.53%)	N/A	N/A	N/A	N/A
Levetiracetam	801 (4.31%)	81 (13.75%)	1.97 (1.35–2.88)	<001	1.83 (1.25–2.69)	0.002
Valproic	80 (0.43%)	15 (2.55%)	3.64 (1.95–6.85)	<001	3.70 (1.96–7.00)	<001
Oxcarbazepine	248 (1.34%)	14 (2.38%)	N/A	N/A	N/A	N/A
Zonisamide	60 (0.32%)	5 (0.85%)	N/A	N/A	N/A	N/A
Carbamazepine	851 (4.58%)	122 (20.71%)	2.79 (1.95–3.99)	<001	2.78 (1.94–3.98)	<001
Clonazepam	1361 (7.33%)	32 (5.43%)	0.46 (0.29–0.73)	<001	0.53 (0.33–0.85)	0.008
Topiramate	237 (1.28%)	10 (1.70%)	N/A	N/A	N/A	N/A
Phenobarbital	46 (0.25%)	3 (0.51%)	N/A	N/A	N/A	N/A
Phenytoin	136 (0.73%)	27 (4.58%)	3.87 (2.32–6.45)	<001	3.33 (1.98–5.58)	<001
Pregabalin	7937 (42.75%)	163 (27.67%)	0.40 (0.29–0.56)	<001	0.47 (0.33–0.66)	<001
Primidone	114 (0.61%)	2 (0.34%)	N/A	N/A	N/A	N/A

**Table 10:** ROR calculation.

	Specific Adverse Events	All other Adverse Events
Specific Drug	A	B
All Other Drugs	C	D
ROR = (A/B)/(C/D)		

## Data Availability

The data presented in this study are available on request from the corresponding author and KIDS due to the inclusion of patient information and ethical concerns.

## Supplementary Materials

The following supporting information can be downloaded at: https://www.mdpi.com/article/10.3390/ph18071013/s1, Table S1: Disproportionality analysis on SOC-based ADEs by each AEDs in Elderly Patients.

## Abbreviations

The following abbreviations are used in this manuscript:

ADE	Adverse Drug Event
AE	Adverse Event
AED	Antiepileptic Drug
AST	Acid-Suppressive Therapy
CI	Confidence Interval
H2RA	Histamine-2 Receptor Antagonist
HR	Hazard Ratio
KIDS	Korean Institute of Drug Safety and Risk Management
NSAIDs	Non-steroidal Anti-inflammatory Drug
OR	Odds Ratio
PPI	Proton Pump Inhibitor
RES	Reticuloendothelial System
ROR	Reporting Odds Ratio
SAE	Serious Adverse Event
SOC	System Organ Class
