# Supplementary material for: Investigation into Safety Profiles of Antiepileptic Drugs and Identification of Predictors for Serious Adverse Events: Insights from National Pharmacovigilance Data
Source: Pharmaceuticals (Basel). 2025 Jul 7;18(7):1013. doi: 10.3390/ph18071013 (PMC12299243; doi:10.3390/ph18071013)
Supplement: Supplementary file 1 [file pharmaceuticals-18-01013-s001.zip › pharmaceuticals-3709840-supplementary.pdf]

Table S1. Disproportionality analysis on SOC-based ADEs by each AEDs in Elderly Patients

**AEDs**	**Non-SAEs**		**SAEs**	
	**ROR (95% CI)**	***P-value***	**ROR (95% CI)**	***P-value***
		**Skin and Appendages Disroder**		
Gabapentin	0.42 (0.37-0.48)	<0.001	0.83 (0.42-1.65)	0.60
Divalproex	2.01 (0.70-5.83)	0.197	N/A	N/A
Lamotrigine	30.73 (23.72-39.83)	<0.001	4.32 (2.24-8.36)	<0.008
Lacosamide	2.49 (1.57-3.96)	<0.001	N/A	N/A
Levetiracetam	3.22 (2.69-3.86)	<0.001	0.19 (0.07-0.53)	0.001
Valproic acids	5.98 (3.75-9.53)	<0.001	N/A	N/A
Oxcarbazepine	7.46 (5.74-9.70)	<0.001	1.69 (0.52-5.49)	0.382
Carbamazepine	6.03 (5.16-7.05)	<0.001	3.55 (2.27-5.56)	<0.001
Clonazepam	0.53 (0.41-0.69)	<0.001	0.76 (0.28-2.02)	0.287
Topiramate	1.31 (0.85-2.00)	0.218	N/A	N/A
Phenobarbital	7.51 (4.14-13.6)	<0.001	N/A	N/A
Phenytoin	**9.18 (6.51-12.94)**	**<0.001**	**3.06 (1.38-6.80)**	**0.006**
Pregabalin	0.27 (0.24-0.31)	<0.001	0.18 (0.09-0.36)	<0.001
		**Musculoskeletal Disorder**		
Gabapentin	1.06 (0.70-1.59)	0.789	N/A	N/A
Levetiracetam	1.13 (0.46-2.79)	0.787	N/A	N/A
Carbamazepine	1.76 (0.85-3.63)	0.126	N/A	N/A
Clonazepam	1.52 (0.81-2.84)	0.194	N/A	N/A
Pregabalin	0.72 (0.48-1.08)	0.11	N/A	N/A
		**Central and Peripheral Nervous System Disorder**		
Gabapentin	1.05 (0.98-1.13)	0.191	1.66 (0.84-3.27)	0.145
Lamotrigine	0.09 (0.04-0.18)	<0.001	N/A	N/A
Lacosamide	0.85 (0.55-1.3)	0.439	N/A	N/A
Levetiracetam	0.27 (0.21-0.35)	<0.001	N/A	N/A
Valproic acids	0.39 (0.19-0.78)	0.008	N/A	N/A
Oxcarbazepine	5.31 (3.67-7.69)	<0.001	N/A	N/A
Zonisamide	0.28 (0.11-0.70)	0.006	N/A	N/A
Carbamazepine	0.75 (0.63-0.89)	<0.001	1.11 (0.49-2.52)	0.811
Clonazepam	0.56 (0.48-0.65)	<0.001	1.04 (0.52-2.05)	0.922
Topiramate	0.66 (0.47-0.92)	0.014	N/A	N/A
Phenytoin	0.56 (0.35-0.89)	0.014	N/A	N/A
Pregabalin	1.59 (1.49-1.70)	<0.001	2.48 (1.51-4.07)	<0..001
Primidone	1.73 (1.18-2.54)	0.005	N/A	N/A
		**Vision Disorders**		
Gabapentin	0.72 (0.48-1.08)	0.116	N/A	N/A
Levetiracetam	0.97 (0.40-2.39)	0.952	N/A	N/A
Topiramate			54.45 (13.23-224.12)	<0.001
Pregabalin	1.15 (0.80-1.65)	0.443	N/A	N/A
		**Pyschiatric Disorders**		
Gabapentin	1.19 (1.08-1.3)	<0.001	N/A	N/A
Divalproex	1.98 (0.80-4.92)	0.14	N/A	N/A
Lamotrigine	0.15 (0.07-0.33)	<0.001	N/A	N/A
Lacosamide	0.80 (0.45-1.43)	0.455	N/A	N/A
Levetiracetam	0.90 (0.71-1.13)	0.382	N/A	N/A

Valproic acids	1.22 (0.66-2.26)	0.52	N/A	N/A
Oxcarbazepine	0.38 (0.22-0.67)	<0.001	N/A	N/A
Zonisamide	1.74 (0.92-3.27)	0.088	N/A	N/A
Carbamazepine	0.49 (0.38-0.65)	<0.001	N/A	N/A
Clonazepam	2.72 (2.39-3.09)	<001	7.18 (2.41-21.40)	<0.001
Topiramate	1.73 (1.25-2.39)	<0.001	N/A	N/A
Phenobarbital	1.04 (0.44-2.45)	0.93	N/A	N/A
Phenytoin	0.37 (0.18-0.8)	0.011	N/A	N/A
Pregabalin	0.70 (0.64-0.77)	<0.001	1.21 (0.45-3.25)	0.699
Primidone	0.97 (0.55-1.70)	0.914	N/A	N/A
**Gastrointestinal Disorders**				
Gabapentin	1.61 (1.40-1.75)	<0.001	N/A	N/A
Divalproex	0.95 (0.33-2.73)	0.92	N/A	N/A
Lamotrigine	0.28 (0.16-0.48)	<0.001	N/A	N/A
Lacosamide	0.42 (0.21-0.83)	0.012	N/A	N/A
Levetiracetam	0.94 (0.77-1.14)	0.516	N/A	N/A
Valproic acids	0.29 (0.11-0.78)	0.014	N/A	N/A
Oxcarbazepine	0.35 (0.21-0.59)	<0.001	N/A	N/A
Zonisamide	0.84 (0.40-1.76)	0.636	N/A	N/A
Carbamazepine	0.45 (0.35-0.58)	<0.001	N/A	N/A
Clonazepam	0.99 (0.85-1.15)	0.903	N/A	N/A
Topiramate	0.76 (0.51-1.12)	0.159	N/A	N/A
Phenobarbital	0.52 (0.19-1.44)	0.207	N/A	N/A
Pregabalin	0.86 (0.79-0.93)	<0.001	N/A	N/A
Primidone	0.82 (0.48-1.42)	0.481	N/A	N/A
**Liver and Biliary Disorders**				
Gabapentin	**0.19 (0.11-0.32)**	**<0.001**	N/A	N/A
Levetiracetam	25.62 (18.79-34.96)	<0.001	N/A	N/A
Valproic acids	5.84 (2.11-16.16)	<0.001	N/A	N/A
Oxcarbazepine	5.34 (2.86-9.96)	<0.001	N/A	N/A
Zonisamide	7.94 (2.85-22.15)	<0.001	N/A	N/A
Carbamazepine	1.04 (0.51-2.11)	0.925	N/A	N/A
Clonazepam	0.97 (0.54-1.74)	0.908	N/A	N/A
Topiramate	1.98 (0.73-5.37)	0.182	N/A	N/A
Phenytoin	7.09 (3.42-14.73)	<0.001	4.50 (1.59-12.81)	0.005
Pregabalin	0.12 (0.07-0.21)	<0.001	N/A	N/A
**Metabolic disorders**				
Gabapentin	1.02 (0.79-1.32)	0.863	N/A	N/A
Levetiracetam	2.85 (1.94-4.20)	<0.001	N/A	N/A
Valproic acids	6.37 (3.96-1022)	<0.001	N/A	N/A
Zonisamide	1.50 (0.93-2.44)	0.098	N/A	N/A
Carbamazepine	0.91 (0.56-1.47)	0.685	N/A	N/A
Topiramate	2.10 (0.98-4.50)	0.056	N/A	N/A
Pregabalin	0.54 (0.42-0.71)	<0.001	N/A	N/A
**Cardiovascular Disorders**				
Gabapentin	1.52 (0.92-2.51)	0.103	N/A	N/A
Clonazepam	1.61 (0.73-3.55)	0.235	N/A	N/A
Pregabalin	0.43 (0.24-0.76)	0.004	N/A	N/A

**Heart Rate and Rhythm Disorders**				
Gabapentin	1.31 (0.76-2.25)	0.324	N/A	N/A
Clonazepam	2.09 (0.83-5.25)	0.118	N/A	N/A
Topiramate	6.15 (2.21-17.16)	<0.001	N/A	N/A
Pregabalin	0.50 (0.28-0.91)	0.023	N/A	N/A
**Vascular (Extracardiac) Disorders**				
Gabapentin	0.98 (0.40-2.44)	0.971	N/A	N/A
Pregabalin	0.74 (0.36-1.65)	0.42	N/A	N/A
**Respiratory Disorders**				
Gabapentin	0.72 (0.67-0.77)	<0.01	0.71 (0.40-1.28)	0.254
Divalproex	0.71 (0.27-1.86)	0.481	N/A	N/A
Lamotrigine	0.44 (0.31-0.63)	<0.001	0.50 (0.23-1.11)	0.087
Lacosamide	1.88 (1.31-2.70)	<0.001	N/A	N/A
Levetiracetam	0.56 (0.46-0.68)	<0.001	1.19 (0.73-1.95)	0.485
Valproic acids	0.44 (0.23-0.86)	0.016	N/A	N/A
Oxcarbazepine	0.51 (0.35-0.73)	<0.001	N/A	N/A
Zonisamide	1.44 (0.84-2.48)	0.19	N/A	N/A
Carbamazepine	0.73 (0.61-0.87)	<0.001	0.65 (0.42-1.03)	0.064
Clonazepam	0.96 (0.84-1.09)	0.524	0.46 (0.19-1.15)	0.096
Topiramate	0.87 (0.64-1.19)	0.381	N/A	N/A
Phenobarbital	0.56 (0.25-1.24)	0.153	N/A	N/A
Phenytoin	0.30 (0.17-0.54)	<0.001	N/A	N/A
Pregabalin	1.68 (1.57-1.80)	<0.001	2.84 (1.95-4.13)	<0.001
Primidone	1.44 (0.97-2.13)	0.074	N/A	N/A
**White Cell and RES disorders**				
Gabapentin	**0.16 (0.07-0.35)**	**<0.001**	N/A	N/A
Levetiracetam	13.53 (8.81-2.08)	<0.001	8.69 (4.05-18.62)	<0.001
Carbamazepine	3.46 (1.92-6.25)	<0.001	N/A	N/A
Clonazepam	0.73 (0.29-1.79)	0.486	N/A	N/A
Topiramate	3.56 (1.30-9.77)	0.014	N/A	N/A
Phenytoin	17.76 (9.00 -35.04)	<0.001	N/A	N/A
Pregabalin	0.93 (0.43-2.02)	0.862	N/A	N/A
**Urinary System Disrorders**				
Gabapentin	**1.80 (1.48-2.19)**	**<0.001**	N/A	N/A
Levetiracetam	0.77 (0.45-1.32)	0.341	N/A	N/A
Phenytoin			13.79 (3.77-50.46)	<0.001
Pregabalin	0.74 (0.60-0.90)	0.003	N/A	N/A
**Body as Whole Disorders**				
Gabapentin	1.33 (1.19-1.49)	<0.001	1.62 (0.84-3.13)	0.15
Lamotrigine	2.89 (1.40-5.93)	0.004	N/A	N/A
Levetiracetam	0.80 (0.59-1.07)	0.134	N/A	N/A
Oxcarbazepine	0.71 (0.27-1.93)	0.506	N/A	N/A
Carbamazepine	0.72 (0.42-1.24)	0.235	N/A	N/A
Clonazepam	0.99 (0.68-1.43)	0.936	N/A	N/A
Topiramate	0.67 (0.37-1.20)	0.178	N/A	N/A
Phenobarbital	1.20 (0.43-3.36)	0.726	N/A	N/A
Phenytoin	0.79 (0.38-1.61)	0.512	N/A	N/A
Pregabalin	0.98 (0.88-1.10)	0.728	0.69 (0.40-1.20)	0.19

Primidone	0.70 (0.31-1.59)	0.395	N/A	N/A
